# Management Impacts on Carbon Dynamics in a Sierra Nevada Mixed Conifer Forest

**DOI:** 10.1371/journal.pone.0150256

**Published:** 2016-02-26

**Authors:** Sabina Dore, Danny L. Fry, Brandon M. Collins, Rodrigo Vargas, Robert A. York, Scott L. Stephens

**Affiliations:** 1 Department of Environmental Science, Policy, and Management, University of California, Berkeley, California, United States of America; 2 United States Department of Agriculture Forest Service, Pacific Southwest Research Station, Davis, California, United States of America; 3 University of California Center for Fire Research and Outreach, College of Natural Resources, University of California, Berkeley, California, United States of America; 4 Department of Plant and Soil Sciences, University of Delaware, Newark, Delaware, United States of America; 5 University of California Berkeley Center for Forestry, Georgetown, California, United States of America; Tennessee State University, UNITED STATES

## Abstract

Forest ecosystems can act as sinks of carbon and thus mitigate anthropogenic carbon emissions. When forests are actively managed, treatments can alter forests carbon dynamics, reducing their sink strength and switching them from sinks to sources of carbon. These effects are generally characterized by fast temporal dynamics. Hence this study monitored for over a decade the impacts of management practices commonly used to reduce fire hazards on the carbon dynamics of mixed-conifer forests in the Sierra Nevada, California, USA. Soil CO_2_ efflux, carbon pools (i.e. soil carbon, litter, fine roots, tree biomass), and radial tree growth were compared among un-manipulated controls, prescribed fire, thinning, thinning followed by fire, and two clear-cut harvested sites. Soil CO_2_ efflux was reduced by both fire and harvesting (ca. 15%). Soil carbon content (upper 15 cm) was not significantly changed by harvest or fire treatments. Fine root biomass was reduced by clear-cut harvest (60–70%) but not by fire, and the litter layer was reduced 80% by clear-cut harvest and 40% by fire. Thinning effects on tree growth and biomass were concentrated in the first year after treatments, whereas fire effects persisted over the seven-year post-treatment period. Over this period, tree radial growth was increased (25%) by thinning and reduced (12%) by fire. After seven years, tree biomass returned to pre-treatment levels in both fire and thinning treatments; however, biomass and productivity decreased 30%-40% compared to controls when thinning was combined with fire. The clear-cut treatment had the strongest impact, reducing ecosystem carbon stocks and delaying the capacity for carbon uptake. We conclude that post-treatment carbon dynamics and ecosystem recovery time varied with intensity and type of treatments. Consequently, management practices can be selected to minimize ecosystem carbon losses while increasing future carbon uptake, resilience to high severity fire, and climate related stresses.

## Introduction

Forest ecosystems constitute a major reservoir of global terrestrial carbon [1–2] and have the potential to sequester anthropogenic carbon emissions. Understanding carbon cycling in forest ecosystems is therefore critical for estimating the global carbon budget. Today most forests have been altered by centuries of human impact. Management practices of timber harvesting, livestock grazing, and fire suppression/exclusion have altered tree density and species composition [3]. In many western North American forests these changes in tree density and species composition, coupled with increases in surface fuel loads, have increased vulnerability to uncharacteristically large, intense fires [4–5]. To reduce this vulnerability and to increase forest resilience, management practices often seek to remove biomass either by mechanical methods or prescribed fire [6]. However, these practices represent a disturbance to forest ecosystems, which reduce photosynthetic active surfaces, remove carbon stored on-site, and alter physical and chemical properties of soils [7]. Even if treatments cause short-term carbon losses, these losses need to be weighed against extended and extensive losses that can be caused by high severity fires [8–10]. It is also necessary to consider other possible benefits of treatments such as enhancement of forest resilience and resistance, increased biodiversity, improvement of hydrological benefits, and erosion protection.

Fuels reduction and harvesting treatments (i.e. prescribed fire, thinning, and clear-cut harvest) can affect ecosystem carbon pools and soil CO_2_ efflux. Soil CO_2_ efflux (*Fs*) is the flux of microbial and plant-respired CO_2_ that represent the largest source of CO_2_ from terrestrial ecosystems to the atmosphere [1]. Thinning and prescribed fire represent partial, or low severity forest disturbances, in that they are not stand-replacing. Partial disturbances are important because they affect more forested area than do stand replacing disturbances [11–12], and because their occurrence is predicted to increase with climate change [8, 13].

Recent literature documenting the effects of thinning and prescribed burning in the southwestern U.S. region integrate the analysis of the effects of disturbances on commercial timber yield with different ecosystem components, including understory vegetation [14], carbon storage, and carbon fluxes [7, 15–20]. These studies show that severe disturbances often switch forests from sinks to sources of carbon [16, 21]. Post-disturbance emission of carbon by decomposition can exceed assimilation of carbon by vegetation for decades [21–22]. In addition, these studies demonstrate that the trajectory and the time needed for ecosystem recovery have implications for long-term and regional scale carbon balances [9, 23]. Therefore, it is important to monitor ecosystems for longer periods (>5 years) to fully capture the implications of management on ecosystem carbon dynamics [12, 22]. The present research is among few studies (see also [23–24]) to include long-term measurements of carbon stocks and dynamics, starting seven years before treatments and continuing for 10 years post-treatment.

This study examines the effects of prescribed fire, thinning, and clear-cut harvesting on ecosystem carbon pools (tree biomass and soil pools), tree radial growth, and *Fs* in a mixed conifer forest in the Sierra Nevada. Our results increase the understanding of forest ecosystem carbon dynamics following management treatments and their dependence on treatment type, intensity and frequency. Our objectives were to: 1) quantify treatment effects on *Fs*. In particular separating the effects of changes in soil temperature and water content from changes in basal rates (*Fs* at a reference soil temperature); 2) compare relationships between *Fs* and soil temperature, CO_2_ concentrations and diffusion through the soil profile across different forest treatments; 3) assess the magnitude of treatment effects on carbon stocks, and their dependence on treatment intensity and type, and time since treatment; 4) quantify effects of thinning and fire on tree radial growth. Our ultimate goal is to inform how forest management practices can be selected to minimize forest carbon losses while increasing future carbon uptake, resilience to high severity fire, and climate related stresses.

## Methods

### Study site

The study was conducted at Blodgett Forest (38°54′N, 120°39′W), a University of California Research Station on the western slopes of the northern Sierra Nevada approximately 21 km east of Georgetown, California. Blodgett is a mixed-conifer forest composed of sugar pine (*Pinus lambertiana*), ponderosa pine (*Pinus ponderosa*), white fir (*Abies concolor*), incense-cedar (*Calocedrus decurrens*), Douglas-fir (*Pseudotsuga menziesii*), and California black oak (*Quercus kelloggii*). Elevation ranges from 1100 to 1410 m. Total annual precipitation averages about 1600 mm, falling mainly between September and May. The average minimum daily temperature in January is 0.6°C and the average maximum daily temperature in July is 28.3°C [25]. The loamy-sandy soils are underlain by Mesozoic, granitic material and are predominantly classified as the Holland and Musick series (fine-loamy, mixed, semiactive, mesic Ultic Haploxeralfs [26]).

The forest is an actively managed timberland and has been repeatedly harvested with both even- and uneven-aged methods. Wildfires have been suppressed at Blodgett in the last century, reflecting a management history common to many forests in California and elsewhere in the western United States [27]. Prior to fire suppression, the median composite fire interval at the 9–15 ha spatial scale was 4.7 years with a range of 4–28 years [28].

### Forest management treatments

This study includes six treatment types: un-manipulated control (CTRL), prescribed fire (FIRE), thinning (THN), thinning plus prescribed fire (THN+FIRE), and clear-cut harvest with and without mechanical soil ripping (HARV_RIP_, HARV_NO_RIP_, respectively). The overall goal of the FIRE and THN practices were to reduce the vulnerability of these forests to high-severity fires [29]. Soil ripping is a common post-harvesting practice used in commercial forests to reduce soil compaction and prepare the soil for seedling planting.

**Fig 1:**
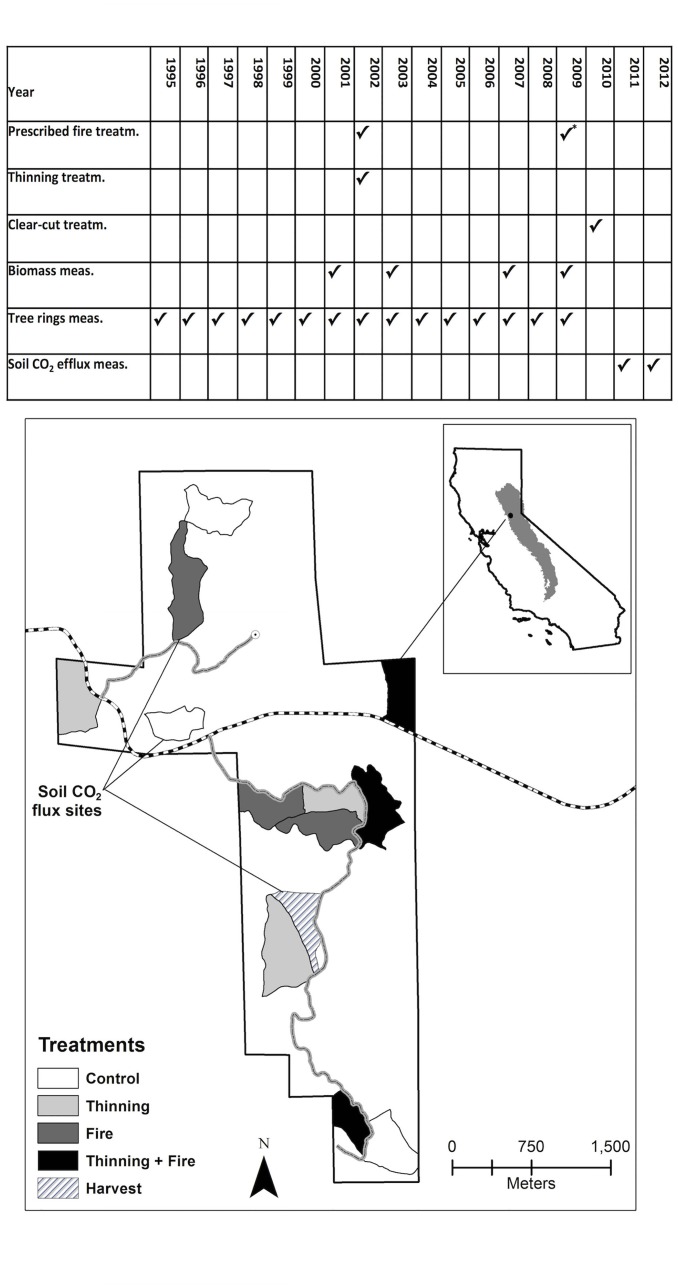
Study layout. Map of the Blodgett Forest. The control, mechanical thinning, prescribed fire, thinning followed by prescribed fire treatments each had three replicates. Soil CO_2_ flux and soil carbon pools were measured in the clear-cut harvest treatment area and one of the replicates of the control and prescribed fire treatments. Timing of treatments (treat.) and measurements (meas.) during a period of 17 years, from 1995 to 2012, is also shown.

The treatments were applied to forest stands with similar climatic conditions, as plots were established within a 10 km radius. Pre-treatment species composition was similar among stands (S1 Table). *Calocedrus decurrens* and *Abies concolor* were the most common species, accounting for about 37% (ranging 34%–40% across treatments) and 27% (ranging 24%–30% across treatments) of the trees, respectively. *Pseudotsuga menziesii* accounted for 13% of the trees (ranging 11%–17% across treatments) and the combination of *Pinus lambertiana* and *Pinus ponderosa* accounted for 8% of the trees (ranging 6%–13% across treatments). The FIRE, CTRL, THN and THN+FIRE treatments were part of the Fire and Fire Surrogate Study (FFS). This study began in 2000 and analyzed the ecological effects of fuel treatments on vegetation structure and other ecosystem characteristics across a network of 13 locations in the United States. Additional information about the study can be found elsewhere [30]. In addition to the FFS treatment sites, we included four sites (each between 2000 and 7000 m^2^) that were clear-cut harvested in the summer of 2010. In these sites all trees were harvested and the residual material was piled and burned. In half of the harvested sites a tractor mechanically ripped soils with a wing-tipped subsoiler. In fall 2010, one-year old ponderosa pine seedlings were planted in pairs, on a 5 x 5 m grid spacing (840 seedlings ha^-1^). For clarity, a schematic diagram showing locations and timing of treatments and measurements is included in Fig 1.

We analyzed tree-ring growth and stand biomass in the CTRL, FIRE, THN and THN+FIRE treatments. Measurements included three replicates per treatment (Fig 1) and continued until 2009, seven years after the prescribed fire and mechanical treatments. Because of practical limitations and instrumentation cost, the analysis on *Fs* and its driving variables (i.e., soil temperature and water content, fine roots, litter and soil carbon) was conducted in one, randomly selected replicate of the CTRL and FIRE treatments (CTRL_Fs_ and FIRE_Fs_) and the newly installed HARV sites (Fig 1). In these four areas soil CO_2_ fluxes, litter, soil carbon, and fine root biomass were measured in 2011 and 2012 (more details on timing in Fig 1).

### Soil CO_2_ efflux measurements

Measurements of *Fs* were taken using the chamber technique [31] and the soil CO_2_ profile technique [32]. At each site (CTRL_Fs_, FIRE_Fs_, and HARV_RIP_ and HARV_NO_RIP_), we periodically measured *Fs* in multiple locations using the chamber technique and continuously monitored *Fs* with the profile technique. Our approach of complementing the chamber and soil CO_2_ profile techniques allowed us to quantify both spatial and temporal variability in *Fs* [33].

#### a. Soil CO_2_ chamber technique

We measured soil CO_2_ efflux using a Li-6000, and later a Li-6400, with a 10 cm diameter soil chamber attachment (Li-Cor, Lincoln, USA) at 110 locations (total for the CTRL_Fs_, FIRE_Fs_, and HARV_RIP_ and HARV_NO_RIP_ sites) bi-monthly during snow free periods (June 2011 to December of 2012). In the spring of 2012 the Li-6000 and Li-6400 were compared in the field obtaining good agreement (r^2^ = 0.95) and therefore, the Li-6000 data were increased for the 15% difference found on the regression slope (data not shown). Measurements across all plots were taken over a two-day period, between 0900 and 1700 hours. The order of the sites and plots measured was randomly changed during each sampling date to avoid a systematic bias based on potential temperature changes. During these manual measurements, the chamber was positioned on PVC soil collars installed 1 cm into the soil to avoid soil disturbance and to allow repeat measurements on the same locations. Soil CO_2_ efflux was calculated from the change of CO_2_ concentration over time and averaged for two cycles over a 10 ppm range encompassing the ambient CO_2_ concentration. Soil water content (*SWC*, integrated over the 0–5 cm depth using a HH2 and ML2x, DeltaT devices, Cambridge, UK) and soil temperature (*Ts*, measured at 10 cm depth using a 6000-09TC, Licor, Lincoln, USA) were measured adjacent to each *Fs* collar.

In both the FIRE_Fs_ and CTRL_Fs_ sites, measurements were collected on 29 different locations per site distributed over 3 ha. In each the HARV_RIP_ and HARV_NO_RIP_ sites, we measured *Fs* in 20 different locations distributed over 6 ha due to the spatial arrangement of the harvest units. More information about measurement location and spatial variability can be found in Dore et al. [34].

#### b. Soil CO_2_ profile technique

We used solid-state CO_2_ sensors (CARBOCAP model GMM 220, range 0–1%, Vaisala, Helsinki, Finland) installed at 2, 8, 16, and 24 cm soil depths to measure *Fs* at each site (CTRL_Fs_, FIRE_Fs_, and HARV_RIP_ and HARV_NO_RIP_). *Ts* was measured with thermocouples at the same depth used for the CO_2_ sensors, and *SWC* (ECH_2_O, Decagon Devices, Pullman, WA, USA) was measured at two depths, 8 and 16 cm. Site specific calibration factors of *SWC* sensors and their dependence on *Ts* were quantified in the lab using each site’s mineral soil sampled over a 20 cm depth. In winter 2012 sensors were calibrated against known CO_2_ concentrations in the lab, and specific parameters determined during calibration were applied to each probe at each site.

CO_2_ concentrations from the sensors were measured every 15 seconds and averaged over 30 minute intervals. The concentrations were corrected for soil temperature and atmospheric pressure and were used to calculate *Fs* (expressed in **μ**mol m^-2^ s^-1^) based on Fick's first law of diffusion *Fs = -Ds dC/dz* [32, 35]. *D*s is the diffusion coefficient in the soil and *C* is the CO_2_ concentration at depth *z*. *Ds* is calculated by the product of the CO_2_ diffusion coefficient in the free air *Da*, and the gas tortuosity factor ξ.

**Fig 2:**
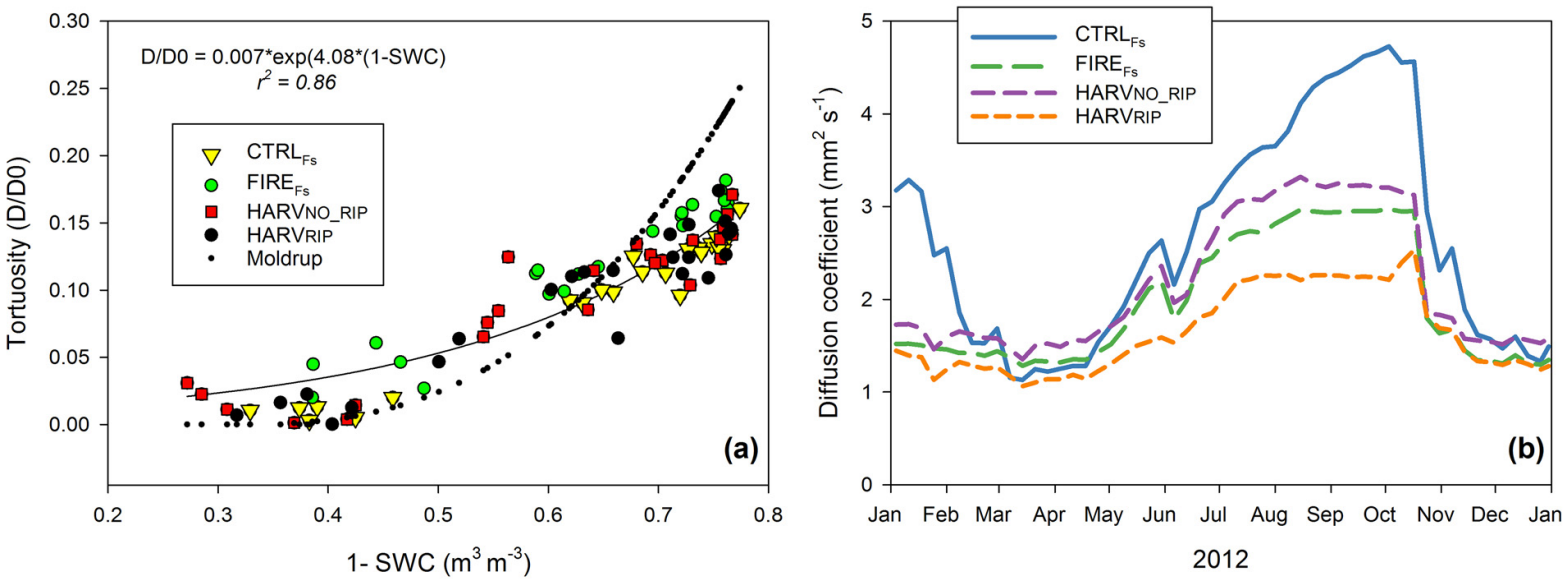
Soil tortuosity and diffusion. (a) Comparison of the empirically determined soil tortuosity factor at the control (CTRL_Fs_), fire (FIRE_Fs_), and tree harvest with (HARV_RIP_) and without soil ripping (HARV_NO_RIP_) treatment sites. Values predicted from the exponential Moldrup curve are included for comparison [35]. (b) Seasonal trend of the diffusion coefficient at the treatment sites calculated from the specific relationship between tortuosity factor and soil water content ξ = 0.007e^4.094^*^(1-swc)^ and the CO_2_ diffusivity in free air, which was affected by soil temperature, atmospheric pressure, and soil water content monitored at each site.

The factor ξ can be quantified empirically by quantifying its relationship with soil porosity (ε), which is determined by the *SWC*, bulk density, and particle density for the mineral soil [35]. We determined site and depth specific ξ in the lab using intact soil samples collected adjacent to the profiles (two replicates per site at 3 depths: 0–5 cm, 10–15 and 15–20 cm; 24 samples total). A sealed 100 ml volume above a 5 cm diameter, 5 cm long aluminum cylinder containing the undisturbed soil sample was initially flushed with nitrogen. Air was able to diffuse from the bottom of the cylinder through the soil. We measured the increase of oxygen with time in the volume above the soil (using a SO-200 Apogee, Logan, USA) for a period of 3–5 minutes after a 2-minute period necessary to reach a constant rate. For each site and depth, measurements were repeated at four different water contents (n = 96 measurements). Following each diffusion measurement *SWC* was determined using the fresh weight of the sample and bulk density obtained from its dry weight (102°C for 24 hours and until constant weight) at the end of all measurements. The resulting equation ξ = 0.0139e^6.2889^*^ε^ (r^2^ = 0.7, p< 0.001) was common to all soils from different sites and depths (Fig 2). To calculate *Fs*, however, we used the relationship between the tortuosity factor ξ and *SWC* (to obtain an exponential trend we used 1- *SWC*). This relationship ξ = 0.007e^4.094^*^(1-swc)^ had a higher coefficient of determination (r^2^ = 0.85, p< 0.001) and was able to quantify the diffusion coefficient independently from accurate site/depth specific measurements of soil bulk density and particle density for the mineral soil. The effects of these soil characteristics were included in the site/depth specific relationship between *SWC* and ξ.

The CO_2_ profile data were adjusted to match the mean spatial values of the chamber data for 2011 and 2012 (S1 Fig). To achieve this, we used for each site and year the slope and intercept of the linear relationships between daily averages of *Fs* simultaneously measured by chambers and each site-profile. Thus, the adjusted values represented the treatment-scale *Fs* and had at the same time high temporal resolution due to continuous measurements. At each site, daily averages of the adjusted *Fs* were fitted with daily averages of *Ts* and *SWC* profiles for 2011 and 2012 using the equation:

$$Fs=S_{10}\cdot Q_{10}{}^{\left(\frac{Ts-10}{10}\right)}e^{-e\left(a-b\cdot swc\right)}$$ (1)

where *S*_*10*_ is basal respiration at 10°C, Q_10_ is the parameter reflecting the temperature sensitivity of *Rs*, and *a* and *b* are the parameters of a simplified Gompertz function [36]. The measurement depth of *Ts* and *SWC* profiles which was able to explain most of the measured seasonal variability in *Fs* was used to parameterize the equation. We used *Ts* at 10 cm at the HARV and FIRE_Fs_ sites and *Ts* at 24 cm at the CTRL_Fs_ site. *SWC* at 8 cm was used at all sites. The fitted equations explained 60% to 85% of the measured *Fs* variability (Table 1).

**Table 1:** Parameters of CO_2_ soil efflux Eq (1), annual total soil CO_2_ emissions, and soil temperature and water content.

		CONTROL	FIRE	HARV_NO_RIP_	HARV_RIP_
**Equation parameters**					
** S**_**10**_	**(g C m**^**-2**^**s**^**-1**^**)**	3.38 *(0*.*07)*	2.58 *(0*.*09)*	2.16 *(0*.*21)*	3.03 *(0*.*22)*
** Q**_**10**_		3.78 *(0*.*18)*	3.44 *(0*.*24)*	2.96 *(0*.*38)*	2.20 *(0*.*43)*
** a**		6.98 *(1*.*96)*	4.58 *(0*.*47)*	7.60 *(1*.*97)*	3.08 *(0*.*81)*
** b**		75.33 *(19*.*13)*	26.62 *(3*.*03)*	48.80 *(12*.*77)*	12.69 *(3*.*71)*
** r**^**2**^		0.86	0.85	0.63	0.61
**Annual soil CO**_**2**_ **efflux**	**(g C m^-2^** **y**^**-1**^**)**				
** 2011**		1180 *(13)*	950 *(13)*	879 *(34)*	666 *(14*)
** 2012**		1309 *(16)*	823 *(15)*	1069 *(48)*	1022 *(20)*
**Soil temperature**	**(°C)**				
** 2011**		8.1 *(0*.*56)*	9.2 *(0*.*68)*	11.9 *(0*.*72)*	13.6 *(0*.*8)*
** 2012**		9.7 *(0*.*55)*	10.7 *(0*.*64)*	12.9 *(0*.*65)*	14.3 *(0*.*82)*
**Soil water content**	**(m**^**3**^ **m**^**-3**^**)**				
** 2011**		0.30 *(0*.*01*)	0.30 *(0*.*01)*	0.32 *(0*.*01)*	0.35 *(0*.*01)*
** 2012**		0.26 *(0*.*01)*	0.29 *(0*.*01)*	0.29 *(0*.*01)*	0.33 *(0*.*01)*

To calculate the annual soil CO_2_ efflux at the control, fire, and harvest rip (HARV_RIP_) and not ripped (HARV_NO_RIP_) sites, the daily profile soil CO_2_ efflux measurements were used to fit a semi-empirical model [36]. *S*_*10*_ is the basal soil CO_2_ efflux at the reference temperature (10°C); *Q*_*10*_ quantifies the temperature dependence of soil CO_2_ efflux; the parameters *a* and *b* are the soil water content dependence of soil CO_2_ efflux. The non-linear regression r^2^ and 95% confidence interval (in parenthesis) of the fitted parameters are also shown. These site-specific parameters, and average daily soil temperature and soil water content were used to calculate annual soil CO_2_ efflux for 2011 and 2012.

Annual fluxes for 2011 and 2012 were obtained by adding daily *Fs* values derived from Eq 1. To calculate uncertainty for annual fluxes at each site we randomly selected 1000 sets of Eq (1) parameters, with each parameter drawn from normal distributions characterized by their fitted means and standard deviation. Each set of parameters was used to compute *Fs* for every day of the year applying the site specific *Ts* and *SWC*. The 1000 annual values calculated from each set of parameter were used to calculate uncertainty as 95% confidence interval.

Effects of treatments on *Fs* were analyzed using different methods. First, we compared chamber fluxes measured on the same day at the different sites, including with this approach both the effect of treatments on basal *Fs* and the effect due to post-treatment differences in *Ts* and *SWC*. Second, we used a general linear model of the form *Fs = f(treatment*, *Ts*, *SWC*) to assess the effect of treatments on *Fs* correcting for the effect of *Ts* and *SWC*, thus removing across-treatment differences in environmental conditions displayed during measurement campaigns. Third, we quantified the treatment effect over the different seasons, comparing the annual sums of *Fs*. To separate how much of the difference in annual fluxes among sites was due to the effects of treatments on *Fs* basal rates and how much was due to changed microclimatic conditions, we modeled *Fs* at the FIRE_Fs_ and HARV sites using the CTRL_Fs_ site’s climatic inputs, as if treatments at these sites didn’t affect environmental conditions but only the *Fs* basal rate.

### Soil and fine root carbon pools

Fine root biomass was measured in summer 2012. Soil samples were collected from 0–15 and 15–30 cm, with a 20 cm^2^ area auger at 10 locations adjacent to randomly selected *Fs* collars at each of the CTRL_Fs_, FIRE_Fs_, HARV_RIP_ and HARV_NO_RIP_ sites (i.e., 20 samples per treatment). Both live and dead fine roots were hand-picked and separated in < 2mm and 2–5 mm diameter classes from each soil core. Fine root dry weight was determined after drying samples at 70°C for 24 hours. The litter layer was collected inside the 10 cm diameter *Fs* collars (27 plots at the FIRE_Fs_ and CTRL_Fs_ sites, 18 plots at both HARV sites) at the end of 2012, and dried at 70°C for 4 days before dividing it into leaves and woody components.

Bulk density of the mineral soil layer (0–5 cm depth) was determined with 5 cm diameter cores collected inside the *Fs* collars (27 plots at both the FIRE_Fs_ and CTRL_Fs_ sites, 18 plots at both the HARV sites) after removing the litter layer, at the end of the *Fs* sampling campaign. Bulk density of the deeper 5 to15 cm soil layer was determined on 8 locations (5 cm diameter, 10 cm long samples), adjacent to randomly selected *Fs* collars at each site. Soil carbon content was determined on 18 samples per site on a 5 cm diameter, 15 cm deep volume. Each sample was separated into two depths, 0–5 cm, and 5–15 cm. Samples were air dried, sieved (2 mm), ground, and finally their carbon content was determined using an elemental analyzer (Thermo Flash 2000, Thermo scientific, MA, USA).

### Tree biomass and annual tree radial growth

Tree biomass and tree radial growth were measured in the CTRL, FIRE, THN, and THN+FIRE treatments. Each treatment had three replicates ranging from 12 to 28 ha (12 experimental plots total). Tree diameter at breast height (DBH), species, and status were measured in 20 circular, 0.04 ha sub-plots distributed on a systematic grid in each experimental plot (240 sub-plots total). Only trees with diameters larger than 5 cm were considered in this study. Measurements of the same plots were collected the year before treatments (2001), the first post-treatment year (2003), and the seventh year post-treatment (2009).

In 2010, in each of the experimental plots, 30–60 trees of the five major species (*Pinus lambertiana*, *Pinus ponderosa*, *Abies concolor*, *Calocedrus decurrens*, *Pseudotsuga menziesii*), distributed over five size classes (DBH < 35 cm, 35–55 cm, 55–75 cm, 75–95 cm, and DBH > 95 cm), were cored and tree-ring widths measured to 0.001mm using a sliding stage to quantify annual growth from 1995 to 2009. The chosen starting point for this analysis was 1995 to have the same length, a seven-year period, before and after treatments.

For each treatment we determined annually individual and mean tree radial growth. The individual tree radial growth is the radial growth of a hypothetical average tree. We calculated it annually for each treatment by averaging the radial growth of each size class and species (i.e. 20 increments per each treatment). The annual mean tree radial growth was calculated by averaging the annual radial growth of all trees present in each treatment. The mean tree radial growth included both the effect of treatments on the growth of each tree (a positive effect in case of reduced competition or a negative effect in case of treatment related injuries) and the effect of decreased tree density (number of surviving trees changing with treatment and year). We used for each tree the mean annual radial growth of its species, experimental plot, and size class. If a size class had no samples, its growth was estimated as the average of the two adjacent classes (same species, year and experimental plot) or as the growth of the closest class (in case of missing smallest or biggest classes). Only 6% of the trees were in size classes with no tree sampled, and of those, 67% had DBH< 35 cm.

We used the allometric equations provided in Jenkins et al. [37] to calculate aboveground tree biomass for each year. For the period 1996 to 2001, diameters were back calculated from all trees initially measured in 2001. We assumed mortality during this period was zero at all sites, as natural cumulative mortality at the CTRL treatment was only 4% between 2001 and 2009. For the period 2002 to 2005 diameters were calculated from all trees remaining after treatments in 2003. For the period 2006–2009 diameters were calculated from the trees recorded in 2009. Coarse root biomass was calculated from aboveground biomass using the equation for temperate forests in Cairns et al. [38]. Aboveground and coarse root biomass was converted to carbon assuming a carbon concentration of 48% [39]. The difference in stand biomass carbon stocks between two consecutive years was used to express the stand annual aboveground tree productivity.

In fall 2010, one-year old ponderosa pine seedlings were planted in pairs on a 5 x 5 m grid in all two HARV sites. To calculate seedling biomass and to analyze the effects of mechanical soil ripping on growth rates, diameter and height of 240 to 400 seedlings were measured in each of the HARV_RIP_ and HARV_NO_RIP_ treatment sites during the summer of 2012. Eighty-five seedlings were collected and dry weight of needles and wood determined. The relationship between diameter and dry weight of needles, wood, and the whole plant was determined separately. The resulting equations were b_L_ = 1.5305 *D*^2.1416^ (r^2^ = 0.73) for needles, b_s_ = 0.9741 *D*^2.2691^ (r^2^ = 0.87) for stems, and b_P_ = 2.5502 *D*^2.1834^ (r^2^ = 0.82) for the whole plant, where *b*_*L*,_ *b*_*S*,_ and *b*_*P*_ was the dry weight of needles, stems, and whole plant, respectively, and *D* was the basal diameter (in cm). The equation for the whole plants was used to calculate the carbon stored as tree biomass at the HARV sites.

### Data Analysis

When *Fs* measured during the same measurement campaign was compared across treatment sites, effects of treatments on *Fs*, *Ts*, and *SWC* were calculated from the slope of the linear relationship between chamber mean at each treatment site against simultaneously measured values at the CTRL_Fs_ site. To evaluate the treatment effects we tested if mean values of *Fs*, *Ts*, and *SWC* at the treatment sites were significantly different using one way repeated measures analysis of variance (ANOVA). When comparing differences of annual *Fs* (and diffusivity) among treatments, we used one-way ANOVA based on daily means. To compare soil carbon, litter, and fine root biomass among CTRL_Fs_, FIRE_Fs_ and HARV sites we used one-way ANOVA.

To quantify the effects of the treatments on tree-ring growth, tree and stand biomass carbon stocks we calculated the effect size using the Before-After Control-Impact (BACI) approach [40]. The BACI effect size = (**μ**ca - **μ**cb)—(**μ**ta - **μ**tb) is the differential change in means (**μ**) between the control (c) and the treatment sites (t) before (b) and after (a) treatment. The effect was evaluated by testing the existence of an interaction between period and treatment using a two-way repeated measures ANOVA and using the seven year averages for pre and post-treatment periods when available. Differences were considered significant at α <0.05 level.

The Tukey *post hoc* test was used to make multiple comparisons among treatments if a significant difference was detected. Normality and equal variance tests were conducted and, if necessary, data were log transformed to meet these conditions. S1 Table summarizes and describes statistical tests used to compare stand characteristic, carbon pools, or fluxes among treatments.

## Results

### Effect of treatments on microclimate

**Fig 3:**
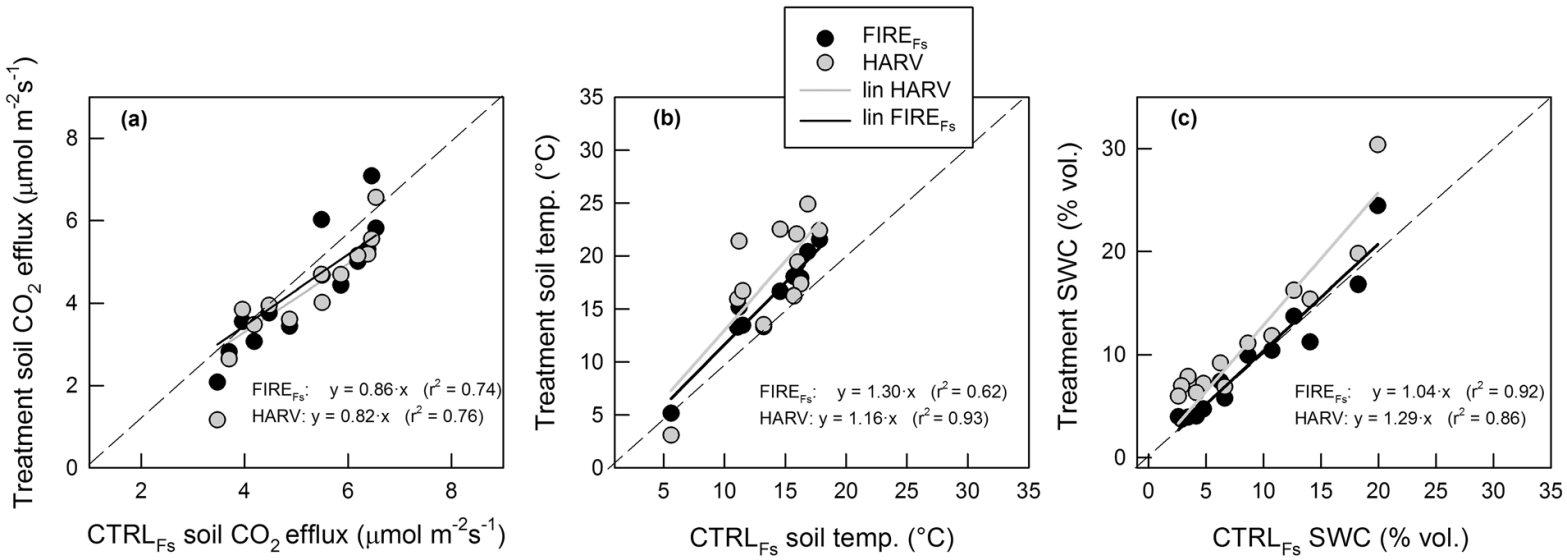
Effects of treatments on soil CO_2_ efflux and soil environmental conditions. Comparison of (a) soil CO_2_ efflux, (b) soil temperature, and (c) soil water content (SWC) between fire (FIRE_Fs_) and harvest (HARV) treatment sites and the control site (CTRL_Fs_). Each symbol represents the average of the 20–29 plots measured in 2012 using the chamber technique. The harvest data is the average of values from units with and without soil ripping. Slope and r^2^ of the linear regression are also shown.

Both fire and tree harvesting reduced tree density of the stands (p < 0.001; Table 1 and S1 Table). Consequently the amount of energy reaching the ground increased, as the direct comparison of *Ts* simultaneously measured at the different sites showed. Soil temperature was 30% higher in the HARV site and 16% higher in the FIRE_Fs_ site (p < 0.001; Fig 3b, S1 Table) compared to the CTRL_Fs_ site. *SWC* was 29% higher at the HARV site and 11% higher at the FIRE_Fs_ site compared to the CTRL_Fs_ site (p < 0.001; Fig 3c, S1 Table). Thus, differences in both *Ts* and *SWC* with the CTRL_Fs_ site were highest at the HARV sites where all vegetation was removed. Seasonal variations of *Ts* and *SWC* were highest at the HARV sites and lowest at the CTRL_Fs_ site.

### Effect of treatments on soil CO_2_ efflux

Soil CO_2_ efflux (measured using the chamber technique) was reduced by treatment (p < 0.001; S1 Table). Comparing *Fs* measured at the different sites on the same measurement date, the FIRE_Fs_ and HARV sites (average of HARV_RIP_ and HARV_NO_RIP_) were 14% and 18% lower, respectively, compared to the CTRL_Fs_ site (Fig 3, S1 Table). Soil CO_2_ efflux was not significantly different between HARV_RIP_ and HARV_NO_RIP_ sites (p > 0.05).

**Fig 4:**
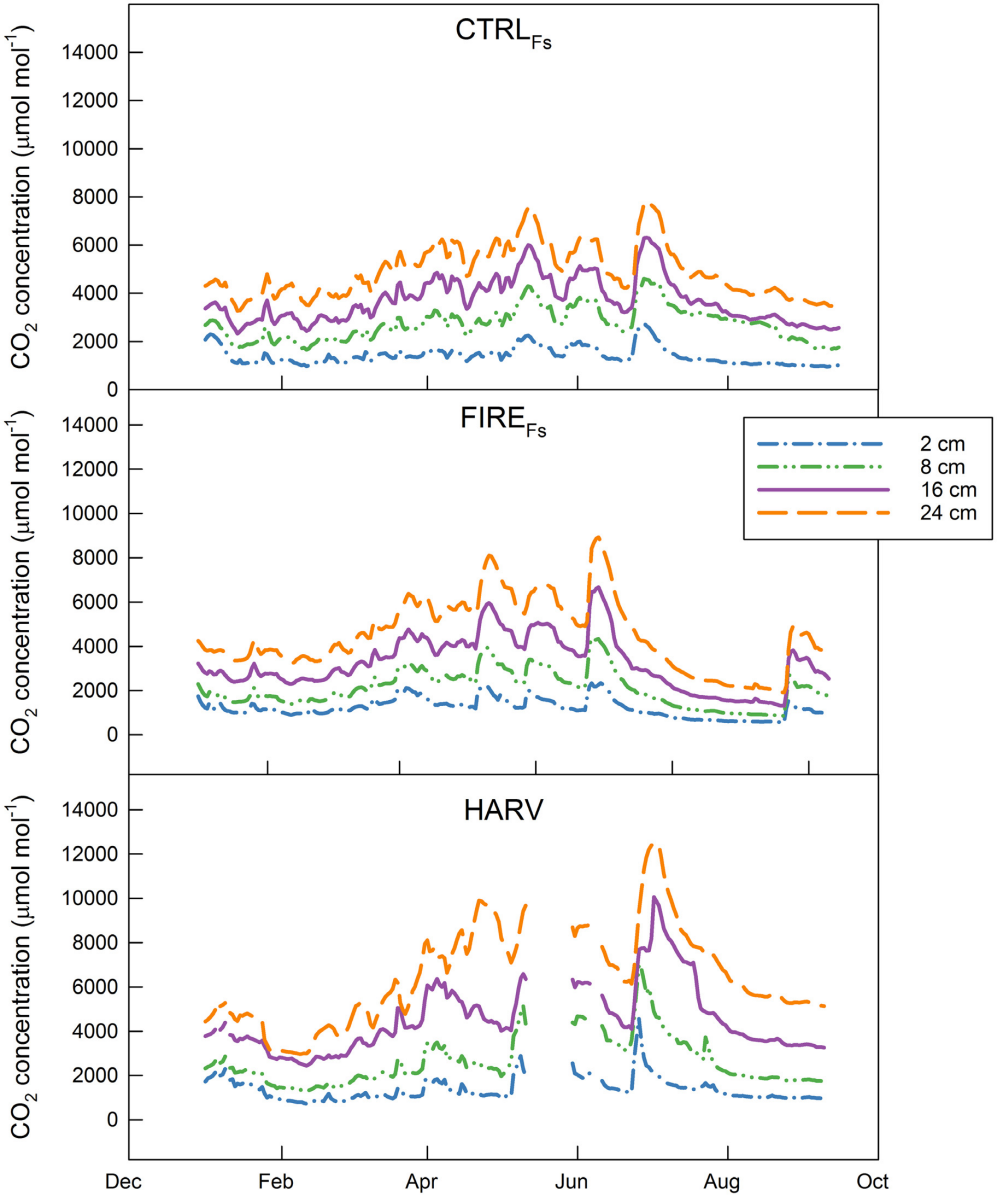
CO_2_ concentration. Concentration of CO_2_ measured at 2, 8, 16 and 24 cm depths at the control (CTRL_Fs_), prescribed fire (FIRE_Fs_), and harvest (HARV) treatment sites in 2012.

CO_2_ concentrations at HARV sites were higher and showed greater seasonal variation (Fig 4) than at the FIRE_Fs_ and CTRL_Fs_ sites, especially at deeper depths (16 and 24 cm). Concentrations of CO_2_ in a soil layer are the result of production and the capacity of CO_2_ to exit the soil (i.e. diffusion). The diffusion coefficient was highest at the CTRL_Fs_ site and lowest at the HARV_RIP_ site (p < 0.001; Fig 2b, S1 Table).

**Fig 5:**
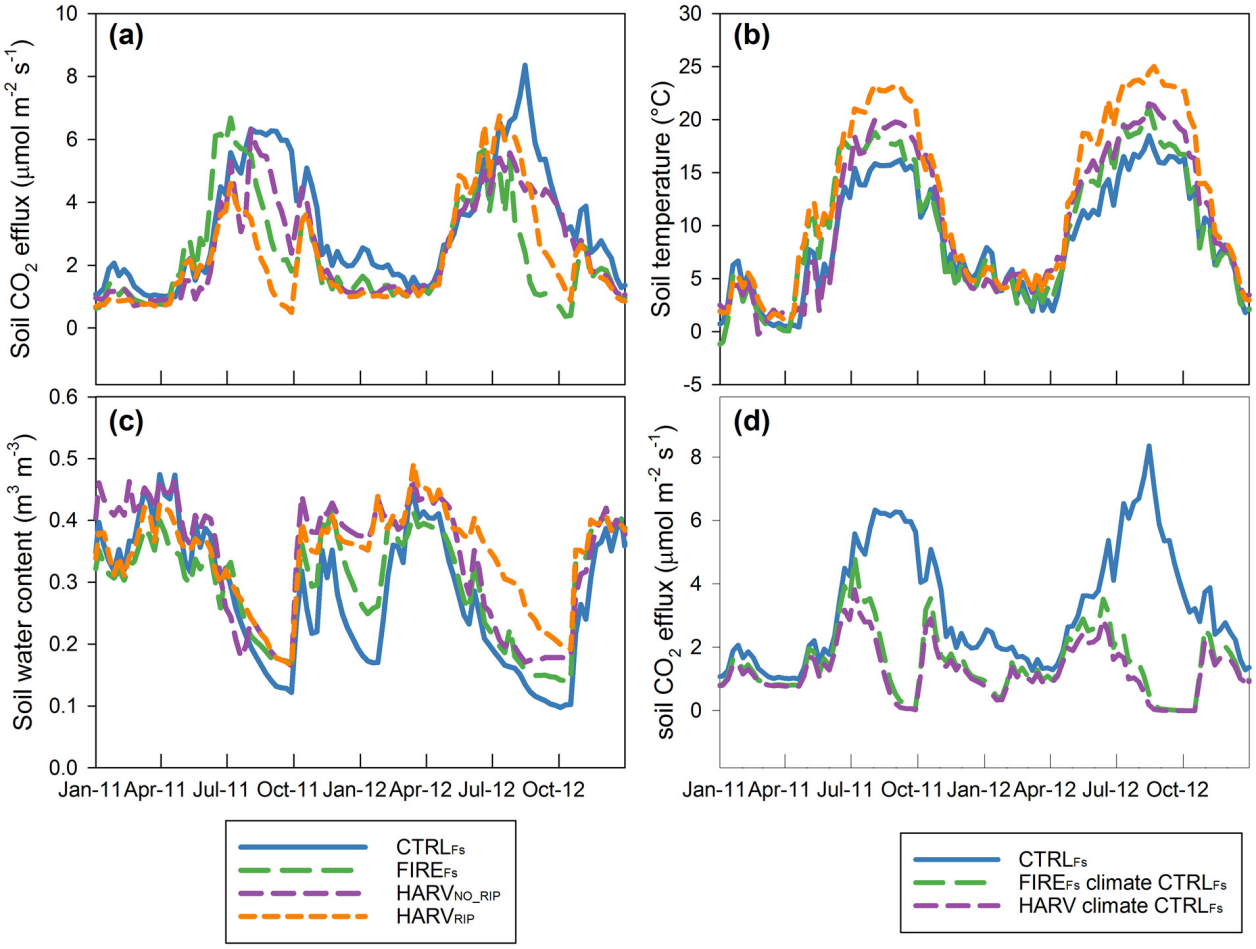
Seasonal trends of soil CO2 efflux, soil temperature and soil water content. (a) Soil CO_2_ efflux, (b) soil temperature (10 cm depth), and (c) soil water content (8 cm depth) recorded during 2011–2012 from profiles installed at the control (CTRL_Fs_), prescribed fire (FIRE_Fs_), and tree harvest with (HARV_RIP_) and without soil ripping (HARV_NO_RIP_) sites. (d) Comparisons of soil CO_2_ efflux at the control site (CTRL_Fs_), and soil CO_2_ efflux at the fire (FIRE_Fs_) and harvest (HARV) sites modeled using climate data recorded at the control site.

FIRE_Fs_ and HARV sites had reduced annual *Fs* compared to the CTRL_Fs_ site (p < 0.001; Table 1 and S1 Table). The annual emissions of treated sites decreased despite having higher *Ts* and *SWC* (Table 1 and S1 Table). Seasonally, sites were more similar during winter and spring, when differences between treated and control site were on average 0.5 **μ**molm^-2^ s^-1^ (Fig 5a) compared to the summer, when difference were 2.5 **μ**molm^-2^ s^-1^. In July-August, when soil water availability decreased (Fig 5c), *Fs* decreased at all sites. Decrease in *Fs* started 2–5 weeks earlier at the treated sites than at the CTRL_Fs_ site for both years (Fig 5a).

### Effect of treatments on soil carbon, fine roots, and litter

Soil carbon (up to a depth of 15 cm) was different among treatment sites when expressed as % content, however not when expressed as g C m^-2^, due to the different soil bulk densities of the sites (p = 0.03; Table 2 and S1 Table). Fine root biomass was similar at the FIRE_Fs_ site compared to the CTRL_Fs_ site (583 and 517 g C m^-2^ respectively), but was reduced by 60–70% (to 143 g C m^-2^ at the HARV_NO_RIP_ and 191 g C m^-2^ at the HARV_RIP_ site) two years after the harvest. The litter layer was reduced at all treatment sites compared to the CTRL_Fs_ site, ranging from -40% in the FIRE_Fs_ (p < 0.008) and HARV_NO_RIP_ sites to –80% in the HARV_RIP_ site (p < 0.001; Table 2 and S1 Table). If we consider carbon stored as mineral soil, fine roots, and litter layer, three years after the second fire (initial 2002, re-burn in 2009, Fig 1), the FIRE_Fs_ site had 19% less carbon than the CTRL_Fs_ site, and two years after the clear-cut the HARV site had 26% less carbon than the CTRL_Fs_ site.

**Table 2:** Soil carbon pools.

Pool (g C m^-2^)		Depth		CTRL_Fs_	FIRE_Fs_		HARV_NO_RIP_	HARV_RIP_	
**Soil**	**0–5 cm**	2435	*(753)*	2453	*(689)*	2866	*(1290)*	2321	*(916)*
	**5–15 cm**	3906	*(577)*	3186	*(364)*	3958	*(1529)*	3025	*(515)*
	**0–15 cm**	6341	*(1330)*	5639	*(1054)*	6824	*(2818)*	5346	*(1432)*
**Fine Roots**	**0–30 cm**	517	*(181)*	583	*(225)*	143	(72) *	191	(157) *
**Litter**	**_**	702	*(155)*	383	*(83)* *	408	(189) *	126	(75) *

Data (g carbon m^-2^) represent averages (and standard deviation) at the control (CTRL_Fs_), prescribed burned (FIRE_Fs_), and harvested sites with and without mechanical soil ripping (HARV_RIP_, HARV_NO_RIP_) for 2012.

Asterisks denote significance (p < 0.05) in the one-way ANOVA applied for comparison of carbon stocks means of treated site and the control site.

### Effects of treatments on forest biomass and tree growth

**Fig 6:**
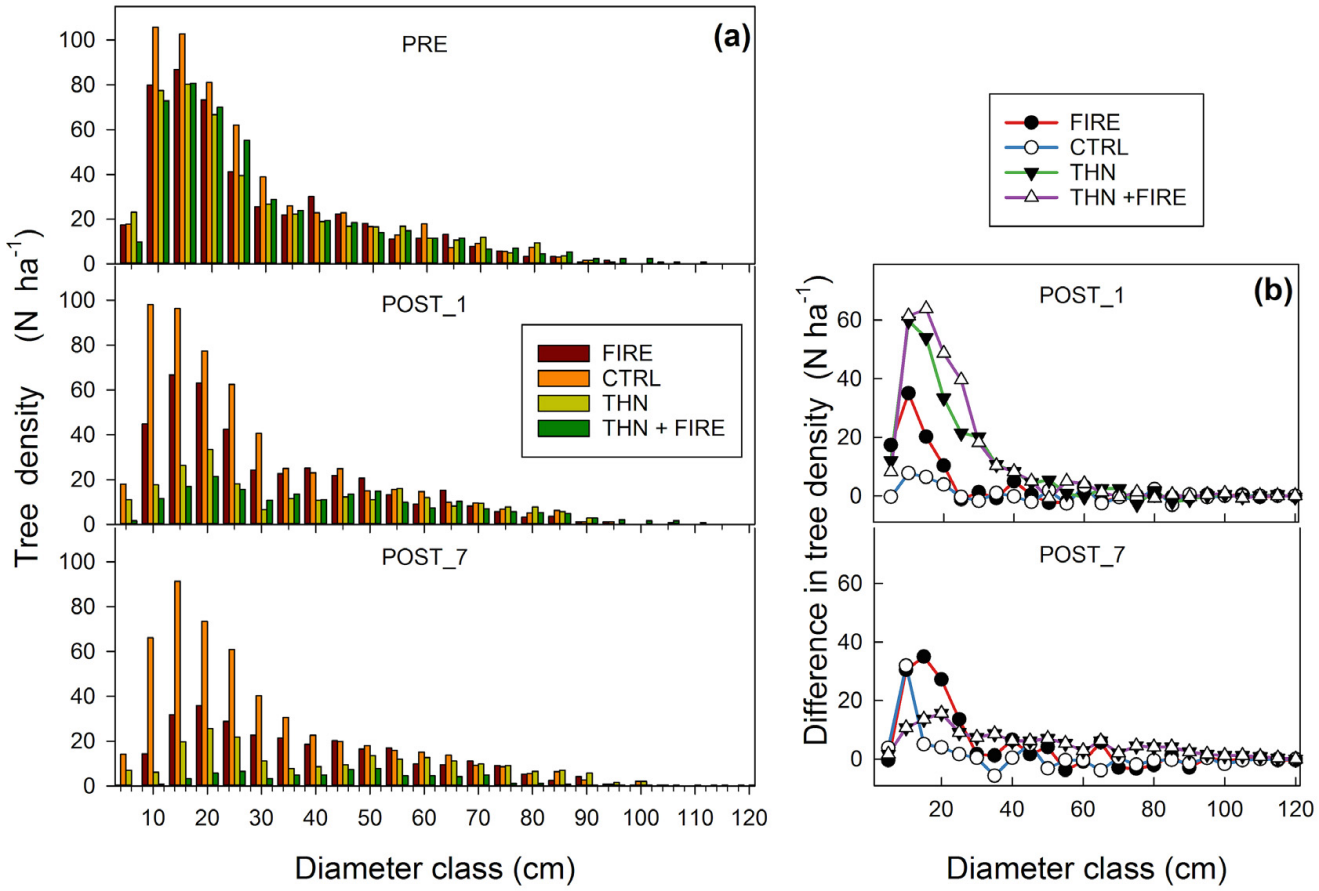
Stand characteristics. (a) Distribution of trees (> 5 cm DBH) in 5 cm diameter classes in the prescribed fire (FIRE), control (CTRL), mechanical thinning (THN) and mechanical thinning followed by prescribed fire (THN+FIRE) treatments at Blodgett Forest (3 replicates per treatment). Tree diameter distribution was measured the year before the treatment (PRE) in 2001, the first post-treatment year in 2003 (POST_1), and seven years post-treatment in 2009 (POST_7). Each class is labeled with the lower interval limit. (b) Difference in number of trees between 2001 and 2003 (POST_1) and 2003 and 2009 (POST_7) by diameter class.

Pre-treatment biomass and tree density did not differ significantly among sites (p >0.05; Table 1 and S1 Table, Fig 6a). All treatments mostly removed or killed small diameter trees (DBH < 30 cm, Fig 6b)

**Fig 7:**
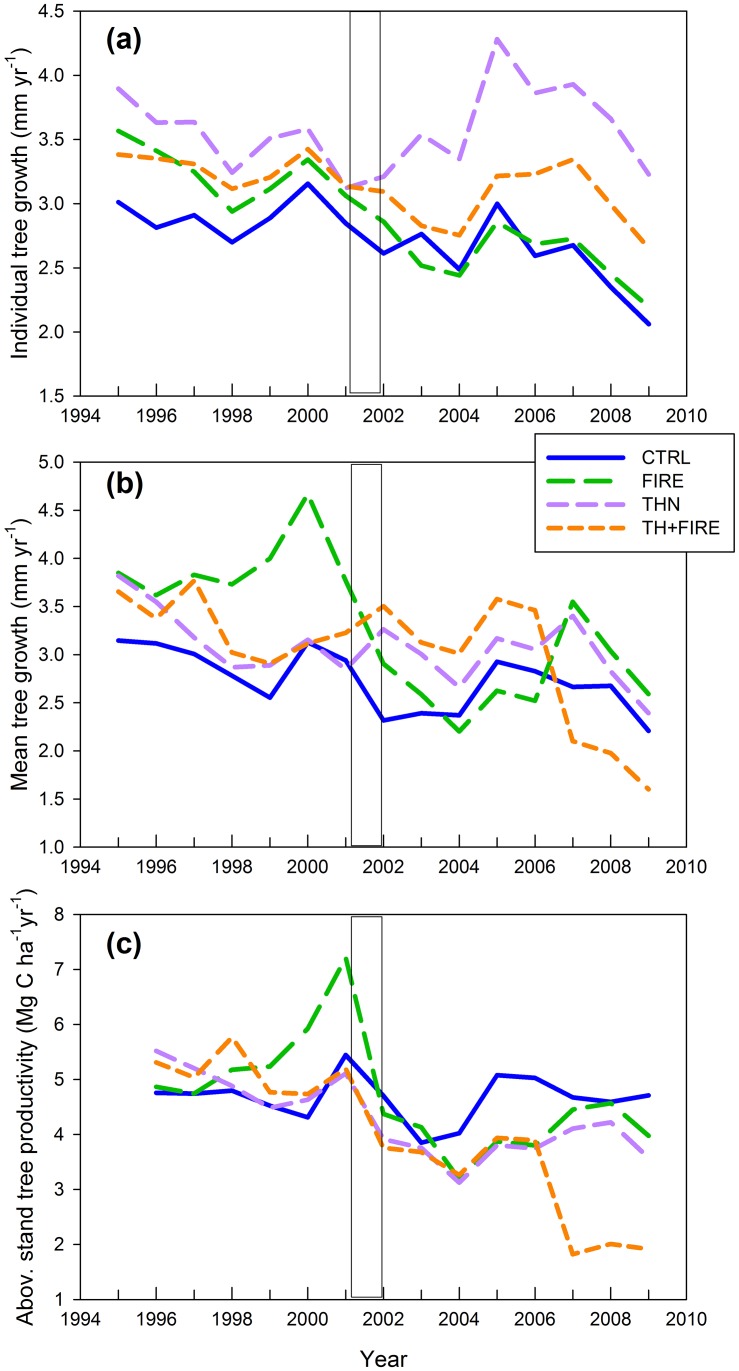
Tree growth and productivity. (a) Annual individual tree radial growth (tree ring widths, in mm yr^-1^) for the control (CTRL), prescribed fire (FIRE), mechanical thinning (THN), and mechanical thinning followed by prescribed fire (THN+FIRE) treatments from 1986 to 2009 at Blodgett Forest. Samples were collected from each of the five tree species equally distributed among size classes. (b) Annual mean tree radial growth (mm yr^-1^), calculated as the average tree growth of all trees in each treatment, identifies both the effect of treatments on tree increment growth (a) and the effect of treatments on tree density. (c) Stand annual productivity was expressed as the difference in stand biomass (aboveground) between two consecutive years. The rectangles show the treatment year 2002. Data are the averages from three replicates.

Over seven years after the treatments, individual tree annual radial growth decreased after FIRE (-10%; p < 0.001), increased 25% after THN (p < 0.001), and was not significantly different after THN+FIRE (p = 0.51 Fig 7a, Table 3 and S1 Table). For each species (*Pinus lambertiana*, *Pinus ponderosa*, *Abies concolor*, *Calocedrus decurrens*, *Pseudotsuga menziesii*) FIRE decreased the annual radial growth by 7–20%, whereas THN increased annual radial growth by 13–30%. The THN+FIRE treatment had a mixed effect, increasing annual radial growth in some species (*Abies concolor* + 33%) but decreasing it in others (*Pseudotsuga menziesi*—13%; Table 3 and S1 Table). Effect of treatments on individual tree annual radial growth depended on tree size. Annual radial growth increased most in smaller diameter trees (<35cm diameter) for all treatments, with the highest increase at the THN treatment (52% increase compared to a 3% decrease at the FIRE treatment, Table 3).

**Table 3:** Tree biomass carbon stocks, growth and productivity.

	CONTROL				FIRE				THIN				THIN + FIRE			
	PRE	POST 1st	POST 7th	Change %	PRE	POST 1st	POST 7th	Effect Size %	PRE	POST 1st	POST 7th	Effect Size %	PRE	POST 1st	POST 7th	Effect Size %
**Biomass**																
** (t C ha**^**-1**^**)**	205 *(37)*	213 *(36)*	240 *(39)*	+14 *	187 *(17)*	187 *(22)*	191 *(23)*	-11 *	208 *(9)*	179 *(8)*	209 *(6)*	-15 *	218 *(25)*	166 *(26)*	170*(28)*	-36 *
**Tree density**																
** (N ha**^**-1**^**)**	558 *(82)*	549 (78)	534 (63)	-4	476 (89)	393 (75)	282 (33)	-32 *	461 (131)	231 (9)	214 (14)	-43 *	465 (51)	191 (4)	155 (14)	-54 *
**Individual tree radial growth**																
** (mm yr**^**-1**^**)**	2.85 *(0*.*53)*		2.06 *(0*.*43)*	-15 *	3.09 *(0*.*31)*		2.20 *(0*.*14)*	-10 *	3.15 *(0*.*07)*		3.29 *(0*.*16)*	+25 *	3.05 *(0*.*21)*		2.57 *(0*.*13)*	+6
** *Abies concolor (27%)***	3.49 *(0*.*19)*		2.80 *(0*.*51)*	-24 *	3.64 *(0*.*21)*		2.71 *(0*.*33)*	-7 *	3.58 *(0*.*30)*		3.71 *(0*.*48)*	+30 *	3.41 *(0*.*10)*		3.66 *(0*.*51)*	+33 *
** *Calocedrus decurrens (37%)***	1.98 *(0*.*22)*		1.90 (0.23)	-4 *	2.06 (0.08)		1.75 (0.13)	-12	2.86 (0.21)		3.00 (0.32)	+13 *	2.14 (0.16)		2.49 (0.34)	+22 *
** *Pinus lambertiana (8%***^***1***^***)***	4.46 *(0*.*29)*		4.09 *(0*.*52)*	-9 *	4.67 *(0*.*32)*		3.96 *(0*.*38)*	-8 *	4.42 *(0*.*37)*		4.92 *(0*.*41)*	+21	4.64 *(0*.*21)*		4.02 *(0*.*30)*	-6
** *Pinus ponderosa (8%***^***1***^***)***	2.72 *(0*.*23)*		2.40 *(0*.*25)*	-13 *	3.62 *(0*.*37)*		2.74 *(0*.*28)*	-19 *	2.65 *(0*.*36)*		2.64 *(0*.*25)*	+13 *	3.25*(0*.*21)*		2.88 *(0*.*45)*	0.4
** *Pseudotsuga menziesii (13%)***	3.70 *(0*.*29)*		3.07 *(0*.*36)*	-20 *	3.62*(0*.*28)*		2.71 *(0*.*41)*	-10 *	3.94 *(0*.*28)*		3.93 *(0*.*49)*	+21	3.84 *(0*.*34)*		2.80 *(0*.*48)*	-13
** Diameter < 35 cm**	*1*.*61 (0*.*11)*		*1*.*55 (0*.*19)*	*-4*	*1*.*81 (0*.*13)*		*1*.*70 (0*.*10)*	*-3*	*2*.*66 (0*.*18)*		*3*.*37 (0*.*33)*	*+52*	*2*.*12 (0*.*11)*		*2*.*30 (0*.*18)*	*+17*
** Diameter 35–55 cm**	*2*.*84 (0*.*13)*		*2*.*46 (0*.*32)*	*-15*	*3*.*34 (0*.*22)*		*2*.*64 (0*.*28)*	*-10*	*3*.*34 (0*.*23)*		*3*.*61 (0*.*34)*	*+29*	*2*.*96 (0*.*12)*		*3*.*15 (0*.*40)*	*+24*
** Diameter 55–75 cm**	*3*.*08 (0*.*15)*		*2*.*72 (0*.*32)*	*-13*	*3*.*53 (0*.*17)*		*2*.*73 (0*.*20)*	*-14*	*4*.*00 (0*.*26)*		*4*.*06 (0*.*43)*	*+19*	*3*.*70 (0*.*15)*		*3*.*38 (0*.*43)*	*+4*
** Diameter >75 cm**	*4*.*32 (0*.*24)*		*3*.*56 (0*.*43)*	*-21*	*4*.*33 (0*.*30)*		*3*.*22 (0*.*38)*	*-10*	*4*.*16 (0*.*39)*		*3*.*85 (0*.*43)*	*+12*	*4*.*10 (0*.*26)*		*2*.*96 (0*.*38)*	*-12*
**Mean tree radial growth**																
** (mm yr**^**-1**^**)**	2.99 *(1*.*54)*		2.24 *(1*.*10)*	-14 *	3.77 *(1*.*01)*		2.59 *(1*.*31)*	-27 *	2.85 *(0*.*04)*		2.41 *(0*.*77)*	+6	3.22 *(0*.*36)*		1.59 *(0*.*30)*	-7
**Stand productivity**																
** (t C ha**^**-1**^ **year**^**-1**^**)**	5.47 *(1*.*33)*		4.77 *(2*.*25)*	-4 *	7.21 *(2*.*82)*		3.97 *(1*.*84)*	-28 *	5.11 *(0*.*59)*		3.59 *(0*.*85)*	-22 *	5.21 *(0*.*55)*		1.91 *(0*.*21)*	-44 *

Comparison of the control, prescribed fire only (FIRE), mechanical thinning only (THIN), and mechanical thinning plus prescribed fire (THIN+FIRE) treatments the year prior to the treatment (PRE = 2001), first year post-treatment (POST 1^st^ = 2003), and seventh year post-treatment (POST 7^th^ = 2009) at Blodgett Forest Research Station in the Sierra Nevada. Biomass is the sum of aboveground and coarse root biomass. Data represent the average (one standard deviation) of 3 replicates. Effects of treatments were expressed as the relative difference between pre-treatment (2001) and seven years after treatments (2009), and were corrected for pre-treatment differences (BACI analysis, Stewart-Oaten, 2001). For radial growth and productivity, pre-post values represent seven-year averages. Radial growth was calculated as average of all species and for single species, as individual radial growth (average of every size class/species) and as mean tree radial growth (average of every tree of each treatment). The average contribution of each species is indicated under its name (%) ^1^value represents the contribution of both pine species.

Asterisks denote significance of treatment effects (p<0.05).

When averaging the tree annual radial growth over the stand, because of the lower post-treatment tree density (Fig 7b), the before-after analysis showed mean annual radial growth decreased 27% after FIRE (p = 0.03) and was not affected by THN (p = 0.4) and THN+FIRE treatments (p = 0.42; Table 3 and S1 Table). All active treatments reduced stand aboveground productivity (expressed as difference between biomass of two consecutive years), however the THN+FIRE treatment had the lowest post-treatment average productivity of 3 t C ha^-1^ (2003–2009), approximately 50% lower than before treatment (p < 0.001; Fig 7c, Table 3).

The CTRL treatment was still accumulating carbon during the observed 14-year period. However, radial growth decreased between the periods 1995–2001 and 2003–2009 from 3.04 (±0.14 SD) to 2.65 (±0.31 SD) mm yr^-1^. From 2001 to 2009, carbon stored as tree biomass increased from 205 (±37 SD) to 240 (±39 SD) t C ha^-1^ at the CTRL treatment, was reduced ca. 10–15% by FIRE (p = 0.005) or THN (p = 0.012), and 36% by the THN+FIRE treatment (p <0.001; Table 3 and S1 Table). All active treatments decreased tree density, however the greatest decrease occurred in the THN+FIRE (54% effect size, p <0.001; Fig 6, Table 3 and S1 Table), and was higher after THN than after FIRE (43% decrease compared to 32% respectively). In the FIRE treatment the number of live trees decreased substantially (> 100 trees ha^-1^, p = < 0.001) between 2003 and 2009. This further decrease between 2003 and 2009 occurred also at the THN+FIRE treatments, compared to a more stable condition at the THN treatment (p = 0.88; Fig 6, Table 3 and S1 Table).

In the first year after treatment, the FIRE treatment did not lose any carbon stored as tree biomass compared to losses of 29 (±0.10 SD) t C ha^-1^ in the THN treatment and the 52 (±0.11 SD) t C ha^-1^at the THN+FIRE treatment (Table 3 and S1 Table). However, between the first and seventh post-treatment years, the FIRE and the THN+FIRE treatments both accumulated a total of ca. 5 (±3 SD) t C ha^-1^ compared to 30 (±3 SD) t C ha^-1^ accumulated by the THN treatment, and 27 (±3 SD) t C ha^-1^ at the CTRL. Both the FIRE and the THN treatments recovered to pre-treatment biomass carbon stocks levels by the seventh post-treatment year (p = 0.13; Table 3 and S1 Table).

At the HARV sites (Table 4), soil ripping did not benefit the seedlings 20 months after planting. No differences were found between HARV_RIP_ and HARV_NO_RIP_ in survivorship, height, and diameter and thus biomass carbon stocks, still ~1 g C m^-2^.

**Table 4:** Characteristics of *Pinus ponderosa* seedlings at Blodgett Forest.

	% Live	Height	Diameter	Needles	Wood	Total Carbon
		(cm)	(cm)	g C m^-2^	g C m^-2^	g C m^-2^
**No rip**	87 *(0*.*05)*	47.8 *(0*.*87)*	3.4 *(0*.*06)*	0.70 *(0*.*06)*	0.53 *(0*.*05)*	1.24 *(0*.*11)*
**Rip**	89 *(0*.*02)*	48.3 *(1*.*05)*	3.3 *(0*.*22)*	0.62 *(0*.*06)*	0.47 *(0*.*05)*	1.10 *(0*.*11)*

Saplings were planted in 2010, with (Rip) and without soil ripping (No rip). Results represent the average (one standard deviation) of two replicated areas for each treatment.

## Discussion

Prescribed fire, thinning, and clear-cut harvest all affected measured pools of carbon in mixed conifer forests in the north-central Sierra Nevada. These treatments also affected ecosystem carbon fluxes, decreasing carbon sequestered annually as tree biomass, or released from the soil. Prescribed fire and thinning affected biomass carbon stocks and growth differently. Over seven post-treatment years, the THN treatment showed a stronger sink strength and reached higher biomass levels compared to treatments that include fire. Our results confirmed that biomass carbon stocks and growth depend on the intensity and type of treatment, and that it is possible to design forest restoration treatments that achieve both increased forest resiliency and carbon storage.

### Treatments and soil CO_2_ efflux

The effect on *Fs* was in part due to the reduction of tree cover, which affected the main drivers of ecosystem processes: energy and water (Table 1 and S1 Table). In addition, forest management treatments altered basal *Fs* (Table 1 and S1 Table), which is related to biotic factors such as soil microbes, root biomass, and soil organic carbon content. The lower basal respiration at the HARV site can be explained by the lack of autotrophic CO_2_ production in the soil.

When we remove the effect of environmental conditions by modeling a hypothetical *Fs* at the FIRE_Fs_ and HARV sites using the CTRL_Fs_ site’s climatic inputs (Fig 5d), we obtained annual *Fs* lower (circa 300 g C m^-2^ year^-1^) than the annual current rates. Thus effects of forest management treatments and effects on microclimate altered soil basal *Fs* in opposite directions: the reduced basal *Fs* lowered the annual *Fs*, but the increase in soil temperature and *SWC* increased *Fs*. Because at our treated sites *Fs* was decreased by treatment (Table 1 and S1 Table), the effect of basal *Fs* was stronger than the effect of microclimate. Complex effects of disturbances on *Fs* were also found in other studies, where factors acted independently on autotrophic and heterotrophic processes and compensatory mechanisms caused disturbances effects in part to cancel out [7, 41].

In several studies analyzing the effects of fire and thinning on *Fs*, researchers also found an increase of soil temperature and soil water content after forest management [17, 41–43]. However, effects on *Fs* varied. Burning increased and thinning reduced *Fs* in forests similar to those studies here [42]. Previous work in young ponderosa pine forests demonstrated that *Fs* generally decreased only when thinning and burning were combined [41, 44]. Misson et al. [7] and Concilio et al. [45] concluded that the response to forest management treatments is proportional to their intensity. Disagreement in results could be due in part to the difficulty to detect treatment effects. In a previous study conducted at our research site we determined that disturbance increased spatial variability of *Fs* [34], mirroring post-disturbance increases in spatial variability of *Ts* and *SWC* and reducing the ability to accurately quantify *Fs* and to detect differences between treatments [34].

Differences in *Fs* among sites were not a result of large differences in maximum and minimum *Fs* rates, but to the timing of their fluctuations. For example, we measured a decline in *Fs* during summer drought, as observed in several other studies [7, 46–48], and treatments changed the onset and duration of these periods (Fig 5a). During the low *SWC* summer periods, the CTRL_Fs_ site showed higher *Fs* than the FIRE_Fs_ and HARV sites (Fig 5a), even if *SWC* was lower at this site (Fig 5b and 5c). This could be due to the difficulties in measuring *SWC* at the appropriate depth, so that the top 5 cm measurement does not represent the layer effectively controlling *Fs*. Summer *SWC* integrated over the root depth could be higher at CTRL_Fs_ than at the treated sites and should be used in future research. An alternative explanation is the prevailing contribution of the autotrophic component at this site, together with an independent control of *Ts* and *SWC* over this component. Tang and others [41] found that the heterotrophic component of *Fs* was more susceptible to seasonal drought than the autotrophic component in a nearby forest.

Our values of Q_10_, ranging from 3.8 (± 0.18 95% CI) to 2.2 (± 0.43 95% CI), showed a decreasing trend going from the CTRL_Fs_, to the FIRE_Fs_ and the HARV sites (Table 1), such that temperature effects were reduced where seasonal temperature variations were higher. Our values matched the values reported by Kobziar et al. [44] in a similar forest located about 100 km south from Blodgett, ranging from 1.9 to 5.38. A lower Q_10_ at the HARV sites, where autotrophic respiration is lowest, agrees with the lower Q_10_ for heterotrophic decomposition than for autotrophic respiration reported in Ngao et al. [36]. Our higher Q_10_ at the CTRL_Fs_ with older trees concurs with the higher Q_10_ for the older stand in a chronosequence of coastal Douglas-fir stands [49]. In our sites Q_10_ was higher at the CTRL_Fs_, where basal respiration was highest, a finding also reported in Davidson et al [46]. Finally Q_10_ decreased at the HARV sites, where temperature was highest, showing a dependence on soil temperature also reported in Tjoelker et al. [50].

We measured seasonal peak daily *Fs* of 6–8 **μ**mol m^-2^ s^-1^ and annual (control treatments) values ranging between 1200 and 1300 g C m^-2^ yr^-1^. Our values are in the range of fluxes measured in previous studies in similar forested ecosystems [25, 41–42, 44]. The relatively high annual *Fs* (for example compared to the range of values for temperate coniferous forests in Subke et al. [51]) was in part explained by a *Fs* activity that persisted late in the fall. Soil temperature was > 10°C until November, and warm and wet conditions are favorable to both heterotrophic and autotrophic processes [46]. Our high winter *Fs* values (2–4 **μ**mol m^-2^ s^-1^) are supported by Xu et al. [25] winter *Fs* values in a ponderosa pine plantation adjacent to Blodgett Forest (3 **μ**mol m^-2^ s^-1^).

The diffusion coefficient of CO_2_ was lower at the HARV and FIRE_Fs_ sites compared to the CTRL_Fs_ site (Fig 2, S1 Table). Differences were not due to modifications in soil physical properties because the relationship between tortuosity and soil water status were not different (Fig 2a). However *SWC* was higher at these sites compared to the CTRL_Fs_ site. Thus soil water content had a prominent role in preventing CO_2_ to exit from the soil surface [46] and could be the cause of the higher CO_2_ concentration recorded at the HARV site (Fig 4). Higher CO_2_ concentration could also be a result of higher CO_2_ production, due to the higher temperature and *SWC* favorable to decomposing processes, or more abundant substrate for decomposition (however we did not find this in terms of soil carbon, litter, or fine roots).

### Fuel Treatment Impacts to Biomass and Tree Growth

After seven years, prescribed fire reduced tree density by 32% and resulted in a 28% reduction in tree biomass productivity and a 10% reduction in individual tree annual radial growth. These reductions were from a combination of direct mortality from fire and secondary mortality [10, 52]. Secondary mortality had a stronger influence on reducing post-fire stand biomass than increased growth rates of the surviving trees, which was also found in other research [17].

Trees that survived the prescribed fire were not able to make efficient use of the increased pool of inorganic nitrogen after fire consumed a large amount of forest floor [53]. A previous related study assessed the impacts of fire and thinning on tree vigor and vulnerability [54] and found negative impacts for fire and positive for thinning. The authors proposed that the negative fire impacts could be minimized by delaying prescribed fire 2–5 years following thinning/mastication, or removing whole-trees instead of mastication which left all activity fuels on site [54].

Over the seven post-disturbance years observed in this study, thinning had a smaller impact on ecosystem carbon dynamics than treatments that included fire. Post-thinning tree density was relatively stable compared to the FIRE treatment. The reduced competition for resources in the THN treatment had a positive impact on tree radial growth on both individual tree and mean tree growth of the treatment. Accumulated carbon was similar than the CTRL treatment both of which were much larger than the FIRE treatment. Nevertheless, if we limited our inventories to the first post-disturbance year, we would have found the opposite conclusion with a stronger impact of thinning than fire (THN reducing biomass carbon stocks 18% and tree density 41%, compared to FIRE reducing biomass carbon stocks 4% and the number of trees by 14%). Fire induced mortality persisted almost a decade, whereas when fuel and biomass were treated mechanically the impacts were concentrated in the first year post treatment.

When analyzing effects of thinning and fire on forests carbon dynamics it is important to include not only the carbon stored in trees, but also the nature and fate of the carbon removed during management operations. With a prescribed fire, most of the carbon removed is released during combustion directly to the atmosphere [8, 10]. Alternatively wood partially burned and standing dead (snags) can take decades to decompose. When biomass is removed by logging, only carbon stored in residual matter that is burned, or in wood used for energy production, is immediately released to the atmosphere. Carbon used as paper and shipping material is stored for longer periods (1–6 years), and for the longest period when wood is used as solid and wood composite products, particularly if used in home construction [55]. Litter, coarse woody debris, and understory vegetation may be only minimally affected by thinning [3, 11, 53]. However carbon is also emitted for transportation of wood products to final destinations and by logging equipment during thinning. At Blodgett forest, thinning did not significantly affect the litter layer or coarse woody debris, whereas both were reduced by prescribed fire [10, 53]. Wood logged by thinning at this forest was sold (with a positive revenue) mostly as construction lumber [55]. Fifty percent of wood removed had a life span of 50 years, and only circa 15% was released to the atmosphere in the first year [10]. In this forest, compared to prescribed fire, thinning caused smaller carbon losses during treatment, kept carbon stored for a longer period, did not remove carbon stored at the soil surface, and increased tree and stand growth.

Despite the decreased productivity and the differences in effects between FIRE and THN treatments, after seven years both of these treatments reached pre-treatment biomass carbon stocks levels, and still maintained a lower fire hazards because of the reduction or removal of surface and ladder fuels [6, 20, 31]. For the THN+FIRE treatment, where 54% of trees were removed or killed, we did not observe any recovery during the first 7 post treatment years. The THN+FIRE treatment displayed the beneficial effect of reduced competition among trees on radial tree growth (also observed at the THN treatment) for two species (*A*. *concolor* and *C*. *decurrens*). However, the positive effects on these species were attenuated with the negative effect caused on other species (especially *P*. *menziesii*).

In comparison with the partial disturbance due to fire or thinning, the clear-cut harvest had the strongest effect on carbon pools and fluxes. Two years after harvesting, carbon stored above ground and as tree biomass was reduced 80% and stand productivity was close to zero. At the same time soil fluxes were reduced by a similar amount as the fire site (ca. 15%). Soil ripping did not have a positive initial effect on seedling growth but it increased the impacts of harvesting on carbon dynamics, as indicated by the larger decrease, compared to the HARV_NO_RIP_ site, of soil carbon and litter.

The intensity of the treatments impacted ecosystem carbon dynamics. A large decrease in tree density (55% reduction) reduced stand biomass carbon stocks and productivity for at least seven years in the thinning followed by fire treatment. When tree density was decreased to a smaller extent (40% reduction) by thinning alone, tree growth was higher than at the untreated stands, and biomass carbon stocks quickly recovered. The intensity of the treatment was not the only determinant factor, the type of disturbance was also important. Fire and thinning, applied singularly or combined, had different impacts on stand productivity and on the time needed to recover to pre-treatment carbon levels. The prescribed fire removed only a relatively small number of trees, with no change in biomass carbon stocks in the first post-treatment year, but tree injuries caused secondary tree mortality and low growth. Therefore, long-term studies are needed to assess the effects of management treatments on carbon dynamics, because studies limited to the first post-disturbance year will not capture the multiple and complex interactions involved. Finally, these results provide insights for managers pursuing forests carbon sequestration to select treatment types and intensities that minimize carbon losses, speed recovery time, and increases post-disturbance carbon uptake.

## Supporting Information

S1 Fig Seasonal trend of soil CO_2_ efflux measured during 2011 and 2012. Soil CO_2_ fluxes measured at the control (CTRL_Fs_), fire (FIRE_Fs_), and tree harvest with (HARV_RIP_) and without soil ripping (HARV_NO_RIP_) treatment sites. Fluxes measured with the profile technique (black line) were adjusted to match fluxes measured periodically at 20–29 locations per site using the chamber technique (black circles).
(TIFF)

S1 Table Statistical analysis. **a)** Description of the statistical analysis of soil carbon pools and CO_2_ efflux (*Fs)* in a mixed conifer forest subject to fire (FIRE), clear cut harvesting with (RIP) and without (NO_RIP) soil ripping, and an undisturbed forest (CTRL) at Blodgett Forest Research Station in the Sierra Nevada. ^1, 2^ compares soil CO_2_ efflux measured using the chamber technique (chamber). ^1^ Test for total effect on *Fs*, where the general linear model ^2^ characterizes interactions among *Fs*, soil temperature, and soil water content. **b**) Statistical analysis of stand characteristics, biomass, growth, and productivity of a mixed conifer forest subject to fire (FIRE), thinning (THN), thinning plus burning (THN+FIRE) and an undisturbed forest (CTRL) at Blodgett Forest Research Station in the north-central Sierra Nevada.
(DOCX)
