# Supplementary figures and images for: Management Impacts on Carbon Dynamics in a Sierra Nevada Mixed Conifer Forest
Source: PLoS One. 2016 Feb 26;11(2):e0150256. doi: 10.1371/journal.pone.0150256 (PMC4769083; doi:10.1371/journal.pone.0150256)

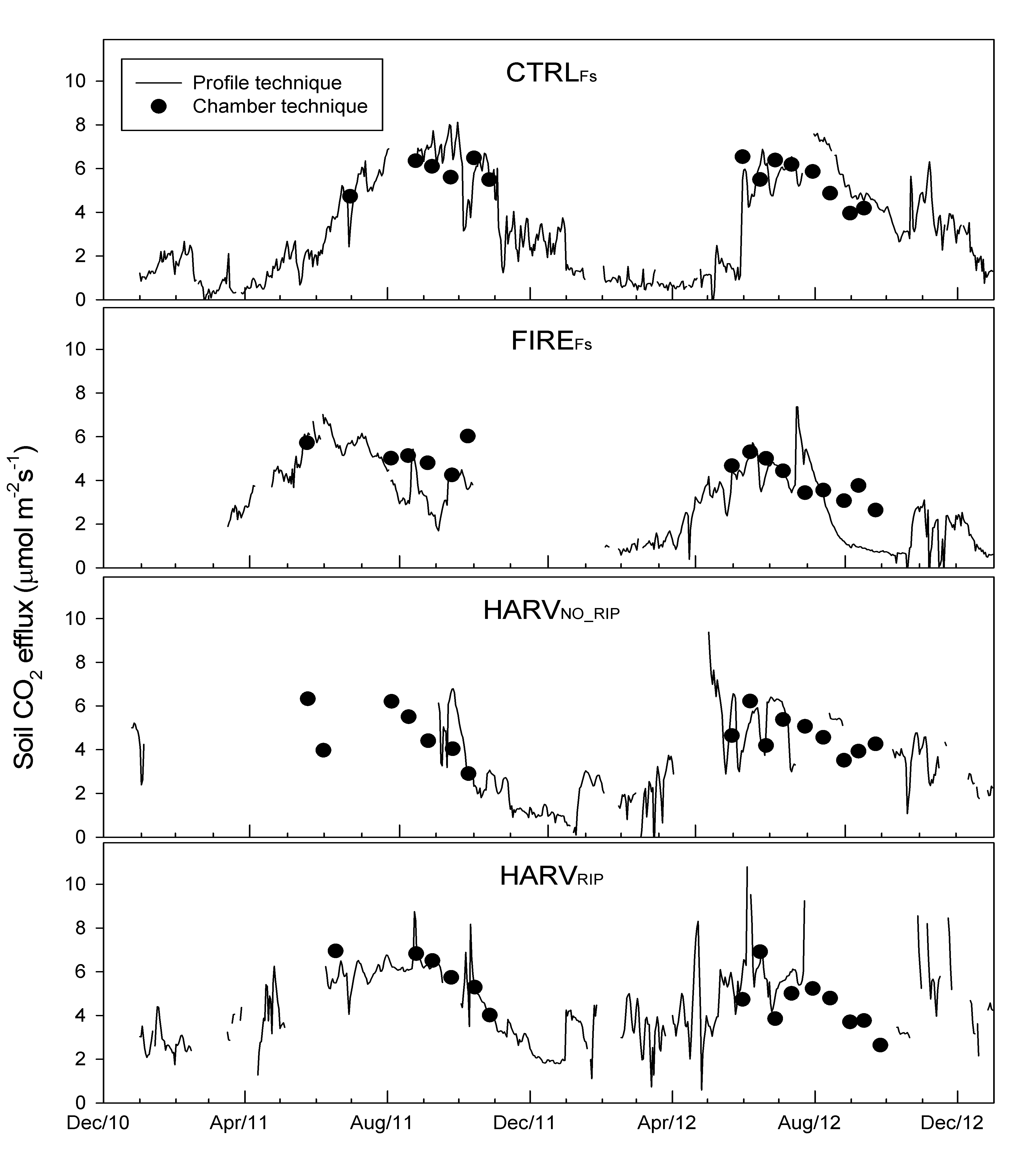

Supplement: S1 Fig — Soil CO2 fluxes measured at the control (CTRLFs), fire (FIREFs), and tree harvest with (HARVRIP) and without soil ripping (HARVNO_RIP) treatment sites. Fluxes measured with the profile technique (black line) were adjusted to match fluxes measured periodically at 20–29 locations per site using the chamber technique (black circles). (TIFF) [file pone.0150256.s001.tiff]
