# Supplementary material for: Management Impacts on Carbon Dynamics in a Sierra Nevada Mixed Conifer Forest
Source: PLoS One. 2016 Feb 26;11(2):e0150256. doi: 10.1371/journal.pone.0150256 (PMC4769083; doi:10.1371/journal.pone.0150256)
Supplement: S1 Table — a) Description of the statistical analysis of soil carbon pools and CO2 efflux (Fs) in a mixed conifer forest subject to fire (FIRE), clear cut harvesting with (RIP) and without (NO_RIP) soil ripping, and an undisturbed forest (CTRL) at Blodgett Forest Research Station in the Sierra Nevada. 1, 2 compares soil CO2 efflux measured using the chamber technique (chamber). 1 Test for total effect on Fs, where the general linear model 2 characterizes interactions among Fs, soil temperature, and soil water content. b) Statistical analysis of stand characteristics, biomass, growth, and productivity of a mixed conifer forest subject to fire (FIRE), thinning (THN), thinning plus burning (THN+FIRE) and an undisturbed forest (CTRL) at Blodgett Forest Research Station in the north-central Sierra Nevada. (DOCX) [file pone.0150256.s002.docx]

**Table S1**

| - **a** |  |  | - **COMPARISON AMONG GROUPS** | | | | | - **MULTIPLE COMPARISON (Tukey)** | | | |
| --- | --- | --- | --- | --- | --- | --- | --- | --- | --- | --- | --- |
|  |  |  |  | |  |  |  | - **CTRL/RIPE** | - **CTR/NO_RIP** | - **CTRL/FIRE** | - **NO_RI/RIP** |
|  |  |  | - **N per site** | | - **df** | - **F** | - **p** | - **p** | - **p** | - **p** | **p** |
| - **Soil carbon (%)** | - **0-5 cm** | - Two way ANOVA | - 16-18 | |  |  |  | - 0.08 | - 0.99 | - **0.029** | - 0.06 |
|  | - **5-15 cm** |  | - 17-18 | |  |  |  | - 0.9 | - 0.404 | - 0.80 | - 0.30 |
|  | - **0-15 cm** |  | - 32-36 | | - 3 | - 3.153 | - **0.030** |  |  |  |  |
| **Soil C** |  | - One way ANOVA | 32-36 | | - 3 | - 0.899 | - 0.446 |  |  |  |  |
| - **Litter** |  | - One way ANOVA | - 20-29 | | - 3 | - 25.356 | - **<0.001** | - **<0.001** | - **<0.001** | - **0.008** | - **0.005** |
| - **Fine root** | - **0-15 cm** | - One way ANOVA |  | |  |  |  |  |  |  |  |
|  | - **15-30 cm** | - One way ANOVA | - 10 | | - 3 | - 12.224 | - **<0.001** | - **0.004** | - **<0.001** | - 0.379 | - 0.811 |
|  | **0-30 cm** | One way ANOVA | - 9-10 | | - 3 | - 11.062 | - **<0.001** | - **0.005** | - **0.011** | - 0.996 | - 0.752 |
|  |  |  | - 9 -10 | | - 3 | - 15.079 | - **<0.001** | - **<0.001** | - **<0.001** | - 0.752 | - 0.999 |
|  |  |  |  | |  |  |  | - **CTRL/FIRE** | - **CTRL/HARV** |  |  |
| - **Soil temperature** |  | One way ANOVA | - 13 | | - 2 | - 12.8 | - **<0.001** | - **0.032** | - **<0.001** |  |  |
| - **Soil water content** |  | One way ANOVA | - 13 | | - 2 | - 19.5 | - **<0.001** | - **<0.001** | - **<0.001** |  |  |
| - **Diffusivity** |  | One way ANOVA | - 365 | | - 3 | - 50.7 | - **<0.001** | - **<0.05** | - **<0.05** |  |  |
| - **Fs annual** |  | One way ANOVA | - 365 | | - 3 | - 22.9 | - **<0.001** | - **<0.001** | - **<0.001** |  |  |
| - **Fs chamber ^1^** |  | One way ANOVA | - 13 | | - 2 | - 15.6 | - **<0.001** | - **<0.001** | - **<0.001** |  |  |
| - **Fs chamber ^2^** |  | GENERAL LINEAR MODEL | - Fs = 0.0607 - (0.941 * treat) + (0.261 * Ts) + (0.134 * SWC) | | | | | | | | |
|  |  |  | - **N** | - **df** | | - **F** | - **p** | - **Rsqr** |  |  |  |
|  |  |  | - 36 | - 3 | | - 13.69 | - **<0.001** | - 0.562 |  |  |  |
|  |  |  |  |  | |  |  | - **Coefficient** | - **value** | - **p** |  |
|  |  |  |  |  | |  |  | - Constant | - 0.0607 | - 0.4 |  |
|  |  |  |  |  | |  |  | - Treat | - -0.941 | - **<0.001** |  |
|  |  |  |  |  | |  |  | - Ts | - 0.261 | - **<0.001** |  |
|  |  |  |  |  | |  |  | - SWC | - 0.134 | - **<0.001** |  |

| - **b** | - **TIMING** | - **TEST** | - **COMPARISON AMONG GROUPS** | | | - **MULTIPLE COMPARISON** - **(Tukey)** | | | | | | | | |
| --- | --- | --- | --- | --- | --- | --- | --- | --- | --- | --- | --- | --- | --- | --- |
|  |  |  |  |  |  | - **CTRL/FIRE** | | | - **CTRL/THN** | | | - **CTRL/THN+FIRE** | | |
|  |  |  | - **df** | - **F** | - **p** | - **df** | - **F** | - **p** | - **df** | - **F** | - **p** | - **df** | - **F** | - **p** |
| - **Biomass** | - Pre | - One-way ANOVA | - 3 | - 0.901 | - 0.48 |  |  |  |  |  |  |  |  |  |
|  | - Post 7 | - One-way ANOVA | - 3 | - 3.495 | - 0.13 |  |  |  |  |  |  |  |  |  |
|  | - Pre vs post7 | - Treat.xTime two way RM. ANOVA | - 3 | - 30.17 | - **<0.001** | - 1 | - 32.15 | - **0.005** | - 1 | - 19.5 | - **0.012** | - 1 | - 92.6 | - **<0.001** |
| - **Species composition** | - Pre | - Treat.xSpecies Two-way ANOVA | - 12 | - 0.382 | - 0.96 |  |  |  |  |  |  |  |  |  |
| **Tree density** | - Pre | One-way ANOVA | - 3 | - 0.739 | - 0.56 |  |  |  |  |  |  |  |  |  |
|  | - Pre vs. post1 | - Time within THN Two-way ANOVA |  |  | - **<0.001** |  |  |  |  |  |  |  |  |  |
|  | - Post1 vs. post7 | - Time within THN Two-way ANOVA |  |  | - 0.88 |  |  |  |  |  |  |  |  |  |
|  | - Pre vs. post1 | - Time within FIRE Two-way ANOVA |  |  | - 0.08 |  |  |  |  |  |  |  |  |  |
|  | - Post1 vs. post7 | - Time within FIRE Two-way ANOVA |  |  | - **0.01** |  |  |  |  |  |  |  |  |  |
|  | - Pre vs post7 | - Treat.xTime two way RM ANOVA | - 3 | - 8.42 | - **0.007** | - 1 | - 24.74 | - **0.008** | - 1 | - 9.16 | - **0.039** | - 1 | - 104.9 | - **<0.001** |
| - **Radial growth** | - Pre vs. Post (7 yrs) | - Treat.xTime two way RM ANOVA | - 3 | - 30.6 | - **<0.001** | - 1 | - 16.96 | - **<0.001** | - 1 | - 19.74 | - **<0.001** | - 1 | - 0.47 | - 0.51 |
| - ***Abies concolor*** | - Pre vs. Post (7 yrs) | - Treat.xTime two way RM ANOVA |  |  |  | - 1 | - 24.75 | - **<0.001** | - 1 | - 15.17 | - **0.002** | - 1 | - 6.67 | - **0.02** |
| - ***Calocedrus decurrens*** | - Pre vs. Post (7 yrs) | - Treat.xTime two way RM ANOVA |  |  |  | - 1 | - 3.93 | - 0.07 | - 1 | - 26.19 | - **<0.001** | - 1 | - 4.70 | - 0.05 |
| - ***Pinus lambertiana*** | - Pre vs. Post (7 yrs) | - Treat.xTime two way RM ANOVA |  |  |  | - 1 | - 4.59 | - 0.05 | - 1 | - 3.31 | - 0.094 | - 1 | - 0.094 | - 0.764 |
| - ***Pinus ponderosa*** | - Pre vs. Post (7 yrs) | - Treat.xTime two way RM ANOVA |  |  |  | - 1 | - 6.16 | - **0.029** | - 1 | - 44.09 | - **<0.001** | - 1 | - 3.82 | - 0.076 |
| - ***Pseudotsuga menziesii*** | - Pre vs. Post (7 yrs) | - Treat.xTime two way RM ANOVA |  |  |  | - 1 | - 10.61 | - **0.007** | - 1 | - 3.00 | - 0.11 | - 1 | - 4.67 | - **0.05** |
| - **Stand radial growth** | - Pre vs. Post (7 yrs) | - Treat.xTime two way RM ANOVA | - 3 | - 4.21 | - **0.012** | - 1 | - 13.8 | - **0.003** | - 1 | - 0.77 | - 0.398 | - **1** | - 0.69 | - 0.422 |
| - **Stand tree productivity** | - Pre vs. Post (7 yrs) | - Treat.xTime two way RM ANOVA | - 3 | - 6.617 | - **0.001** | - 1 | - 13.04 | - **0.004** | - **1** | - 17.6 | - **0.002** | - **1** | - 18.05 | - **0.001** |
